# Artificial Intelligence in Small-Molecule Drug Discovery: A Critical Review of Methods, Applications, and Real-World Outcomes

**DOI:** 10.3390/ph18091271

**Published:** 2025-08-26

**Authors:** Sarfaraz K. Niazi

**Affiliations:** College of Pharmacy, University of Illinois, Chicago, IL 60612, USA; sniazi3@uic.edu

**Keywords:** artificial intelligence, drug discovery, machine learning, deep learning, molecular design, ADMET prediction, virtual screening, diffusion models, agentic AI, foundation models

## Abstract

Artificial intelligence (AI) is emerging as a valuable complementary tool in small-molecule drug discovery, augmenting traditional methodologies rather than replacing them. This review examines the evolution of AI from early rule-based systems to advanced deep learning, generative models, diffusion models, and autonomous agentic AI systems, highlighting their applications in target identification, hit discovery, lead optimization, and safety prediction. We present both successes and failures to provide a balanced perspective. Notable achievements include baricitinib (BenevolentAI/Eli Lilly, an existing drug repurposed through AI-assisted analysis for COVID-19 and rheumatoid arthritis), halicin (MIT, preclinical antibiotic), DSP-1181 (Exscientia, discontinued after Phase I), and ISM001-055/rentosertib (Insilico Medicine, positive Phase IIa results). However, several AI-assisted compounds have also faced challenges in clinical development. DSP-1181 was discontinued after Phase I, despite a favorable safety profile, highlighting that the acceleration of discovery timelines by AI does not guarantee clinical success. Despite progress, challenges such as data quality, model interpretability, regulatory hurdles, and ethical concerns persist. We provide practical insights for integrating AI into drug discovery workflows, emphasizing hybrid human-AI approaches and the emergence of agentic AI systems that can autonomously navigate discovery pipelines. A critical evaluation of current limitations and future opportunities reveals that while AI offers significant potential as a complementary technology, realistic expectations and careful implementation are crucial for delivering innovative therapeutics.

## 1. Introduction

The pharmaceutical industry is experiencing a significant transformation through the integration of artificial intelligence (AI) with traditional drug discovery methodologies. This evolution represents not a replacement of established approaches but rather the development of complementary tools that augment human expertise and computational chemistry methods that have been refined over decades [1,2]. Recent developments underscore this complementary nature: Insilico Medicine’s TNIK inhibitor, INS018_055, created using generative AI in conjunction with traditional medicinal chemistry approaches, progressed from target discovery to Phase II clinical trials in approximately 18 months, demonstrating how AI can accelerate specific aspects of drug development when integrated with conventional methods [3]. This achievement, alongside the growing pipeline of AI-assisted molecules entering clinical trials, signals an evolution in pharmaceutical research where artificial intelligence approaches work alongside established methodologies to address specific challenges in the discovery process [4].

It is crucial to acknowledge that AI represents an additional tool in the drug discovery toolkit rather than a paradigm shift that renders traditional methods obsolete. The success of AI applications depends heavily on the quality of training data, the expertise of medicinal chemists interpreting results, and the robustness of experimental validation—all elements rooted in traditional drug discovery practices. While AI can process vast chemical spaces and identify patterns beyond human capability, it cannot replace the intuition, creativity, and contextual understanding that experienced drug discovery scientists bring to the process.

The transformation extends to regulatory agencies, with the FDA now utilizing AI systems to support the review of drug applications while maintaining traditional review processes [5]. The FDA published a draft guidance in 2025 titled “Considerations for the Use of Artificial Intelligence to Support Regulatory Decision Making for Drug and Biological Products,” informed by over 500 submissions with AI components from 2016 to 2023. The agency’s integration of AI/ML technologies reflects a recognition that these tools can enhance rather than replace traditional regulatory science [6]. As highlighted in a recent Nature Machine Intelligence perspective, this draft guidance establishes a risk-based credibility assessment framework for AI applications that complements existing regulatory frameworks [7].

The convergence of computational power, sophisticated algorithms, and vast biomedical datasets has created opportunities to address specific challenges in pharmaceutical development, where traditional approaches face mounting costs exceeding $2.6 billion per approved drug and development timelines stretching 10–15 years [8]. However, as noted in Nature Machine Intelligence, while deep learning shows promise for drug discovery, including advanced image analysis and molecular property prediction, it requires careful validation. It should be viewed as complementary to established methods rather than a replacement [9]. The dual-use potential of these technologies also raises important ethical considerations, as AI technologies for drug discovery could be misused for de novo design of biochemical weapons [10].

The application of AI in molecular biosimilarity assessment demonstrates how these technologies can enhance rather than replace traditional analytical methods in pharmaceutical development [11]. The success of AI-assisted drug repurposing, such as baricitinib identified through AI analysis for COVID-19 treatment, and the advancement of AI-designed molecules like rentosertib through clinical trials, validates the potential of these approaches when integrated adequately with traditional drug discovery processes. As we evaluate this technological integration, it is essential to maintain realistic expectations about AI’s role as a powerful tool that complements, rather than replaces, the established foundations of pharmaceutical research and development.

### Small Molecules in the Context of AI-Assisted Discovery

Small molecules represent the largest class of approved therapeutics, comprising approximately 90% of all marketed drugs [12]. These compounds offer distinct advantages, including oral bioavailability, tissue penetration, and well-established manufacturing processes. The traditional drug discovery paradigm, while successful in delivering thousands of approved medicines, faces mounting pressures from increasing research and development costs, declining productivity, and stringent regulatory requirements. The pharmaceutical industry’s productivity challenges, often referred to as “Eroom’s Law,” describe the observation that drug discovery efficiency has declined over the past decades, with the number of new drugs approved per billion dollars spent on research halving approximately every nine years [13].

The complexity of modern drug discovery encompasses multiple interconnected challenges. Target validation requires a comprehensive understanding of disease biology and the mechanism of action [14]. Hit identification from millions of potential compounds demands efficient screening strategies [15]. Lead optimization involves simultaneous improvement of multiple properties, including potency, selectivity, pharmacokinetics, and safety [16]. Each stage is characterized by high attrition rates, with only 1 in 5000 discovered compounds ultimately reaching market approval [17]. The traditional drug discovery process costs over $2.6 billion per approved drug and requires 10–15 years from initial discovery to market approval [8].

Artificial intelligence has emerged as a complementary technology that addresses specific challenges by leveraging computational power to process vast chemical spaces, predict molecular properties, and guide experimental design [18,19,20]. Unlike traditional computational approaches that rely on mechanistic models, AI systems can learn complex patterns from large datasets and make predictions for novel chemical entities. However, AI’s effectiveness is limited by data biases and generalizability issues, as highlighted in recent meta-analyses that show variable performance across datasets [21].

Generative AI, a subset of artificial intelligence that creates new content rather than simply analyzing existing data, has become particularly valuable as a tool in drug discovery by enabling the de novo design of novel molecular structures to complement traditional medicinal chemistry approaches [22,23]. Graph Neural Networks (GNNs) represent another crucial advancement, specifically designed to process molecular structures represented as mathematical graphs, where atoms serve as nodes and bonds as edges [24,25]. Convolutional Neural Networks (CNNs), initially developed for image processing, have been adapted for molecular property prediction by treating chemical structures as images or 3D objects [26,27].

The integration of AI into drug discovery workflows has accelerated significantly in recent years, driven by advances in deep learning [28], increased data availability [29], and improvements in computational infrastructure. Key advances in deep generative models [30,31], diffusion models [32], active learning strategies [33], multi-objective optimization [34], and agentic AI systems [35] have demonstrated significant potential to enhance pharmaceutical research and development when used in conjunction with traditional methods. However, critical evaluations reveal limitations in out-of-distribution performance and the need for careful validation.

## 2. Historical Evolution of Computational Small-Molecule Discovery

The application of computational methods to small-molecule drug discovery has evolved through distinct phases, each building upon previous advances while establishing parallel tracks of development rather than linear progression. Early efforts in the 1960s focused on developing quantitative structure-activity relationship (QSAR) models that correlated molecular descriptors with biological activity [36]. These linear models, while limited in scope, established the fundamental principle that molecular structure determines biological function and can be predicted computationally. However, QSAR’s assumptions of linearity often fail in complex biological systems, leading to poor predictions for novel scaffolds [37].

The 1980s witnessed the emergence of structure-based drug design, enabled by advances in computational chemistry and molecular modeling [38]. The development of the DOCK program by Kuntz and colleagues at UCSF represented a seminal achievement, enabling the first systematic approaches to molecular docking [39]. This period also witnessed significant advances in molecular dynamics simulations and force field development, with programs such as AMBER and CHARMM providing tools to model protein-ligand interactions with increasing accuracy. These developments enabled researchers to predict how small molecules interact with biological targets, revolutionizing the rational design of inhibitors. Simultaneously, combinatorial chemistry approaches enabled the synthesis of large compound libraries, creating new opportunities for high-throughput screening campaigns [40].

A significant development during this period was the emergence of pharmacophore-based drug design, which provided an indirect approach to drug discovery when target structures were unavailable. Pharmacophore modeling enabled researchers to define the essential features required for biological activity based on known active compounds, allowing for virtual screening without requiring detailed structural information about the target [41]. This approach proved particularly valuable for targets where obtaining crystallographic structures was complex.

The 1990s marked the transition toward knowledge-based systems and expert systems that encoded medicinal chemistry expertise into rule-based algorithms. These systems, exemplified by programs such as LHASA (Logic and Heuristics Applied to Synthetic Analysis), aimed to automate retrosynthetic planning and predict synthetic feasibility, thereby supporting drug discovery by helping chemists plan efficient synthetic routes to target molecules [42,43]. While LHASA was not directly used for drug design, it played a crucial supporting role in making drug discovery more efficient by predicting the synthetic accessibility of proposed compounds.

The machine learning era (2000s–2015) introduced data-driven approaches that could learn patterns from experimental data without explicit programming. Support vector machines, random forests, and early neural networks began to complement traditional QSAR models, offering improved accuracy and the ability to handle non-linear relationships [44,45]. Importantly, these methods did not replace traditional approaches but rather provided additional tools that could be used in conjunction with structure-based design, pharmacophore modeling, and molecular dynamics simulations. The pharmaceutical industry began generating larger datasets through high-throughput screening, creating opportunities for more sophisticated modeling approaches [46]. Yet, these models often suffered from overfitting and limited interpretability.

The deep learning revolution (2015–2020) fundamentally expanded the computational toolkit available to drug discovery scientists. Deep neural networks demonstrated superior performance in molecular property prediction [47], chemical synthesis planning [30], and drug-target interaction modeling. The development of specialized architectures for molecular data, including graph neural networks [48,49] and molecular transformers [50], has enabled AI systems to learn directly from chemical structure representations. Companies like Atomwise (2012), BenevolentAI (2013), Exscientia (2012), and Insilico Medicine (2014) pioneered the commercial application of deep learning to drug discovery, achieving notable successes [51]. However, critics note that many “successes” are retrospective, with prospective validation rarer [21].

**Figure 1:**
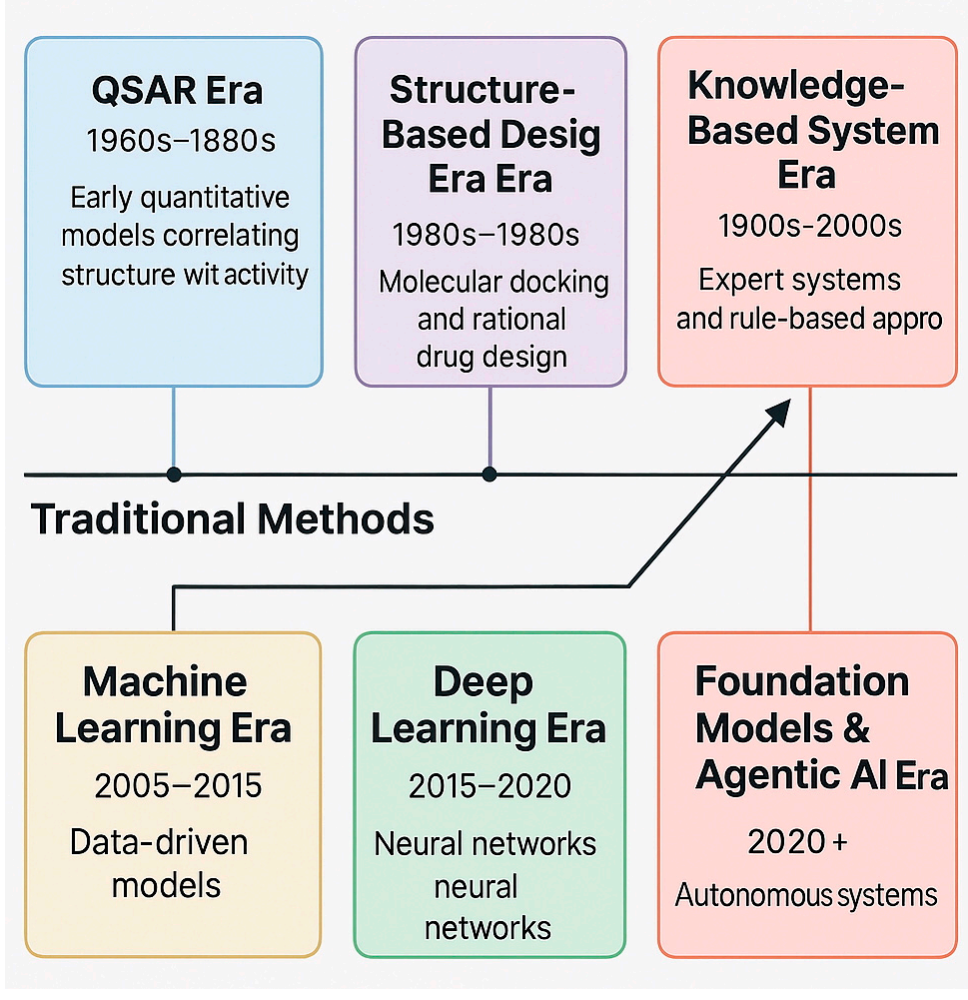
Evolution of Computational Drug Discovery: Parallel Development of Traditional and AI Methods. The timeline illustrates how AI approaches have evolved alongside and complemented traditional computational methods, rather than replacing them. Conventional methods (shown in the blue track) continue to be refined and utilized, while AI methods (shown in the green track) offer additional capabilities. Arrows indicate points of integration and mutual enhancement between the two approaches.

The coexistence and integration of multiple computational approaches characterize the current era (2020-present). Foundation models, diffusion models, and agentic AI systems work alongside traditional molecular dynamics, docking, and QSAR methods. Rather than representing a linear evolution where newer methods replace older ones, the field has developed into a rich ecosystem where different approaches are selected based on the specific problem, available data, and project constraints [35,52]. This timeline illustrates how computational power, algorithm sophistication, and data availability have created an increasingly diverse toolkit for small-molecule discovery rather than a single dominant methodology [13,20] (Figure 1).

## 3. Core Applications of AI in Small-Molecule Discovery

### 3.1. Target Identification and Validation

Target identification represents the earliest stage of drug discovery, where researchers seek to identify biological molecules whose modulation by small molecules can produce therapeutic benefits. Traditional approaches rely on literature review, genetic studies, and phenotypic screening, often requiring years of investigation [14]. AI has enhanced this process by enabling systematic analysis of multi-omics datasets, including genomics, transcriptomics, and metabolomics data to identify targets amenable to small-molecule intervention [53]. However, AI models can perpetuate biases in omics data, such as underrepresentation of non-Western populations, leading to inequitable target selection [54].

Natural language processing (NLP) technologies have proven particularly valuable for target identification by mining the vast biomedical literature. Modern NLP systems can process millions of scientific papers, patents, and clinical reports to identify novel target-disease associations, small-molecule drug repurposing opportunities, and biomarker candidates [55]. IBM Watson for Drug Discovery and similar platforms have demonstrated the ability to generate testable hypotheses by analyzing unstructured text data and identifying hidden connections between genes, diseases, and small-molecule compounds [56]. A critical evaluation reveals that NLP accuracy varies, with false positives resulting from the use of ambiguous language.

Graph-based machine learning approaches have shown promise in target identification by modeling complex biological networks. These methods can integrate metabolic pathways and gene regulatory networks to predict novel therapeutic targets suitable for small-molecule intervention [57]. Knowledge graphs that combine structured databases with literature-derived relationships enable systematic exploration of the target space and identification of previously unexplored therapeutic opportunities for small-molecule drugs [58].

### 3.2. Hit Discovery and Virtual Screening

Hit discovery involves identifying small-molecule compounds that demonstrate measurable activity against a validated target, typically through high-throughput screening of compound libraries containing hundreds of thousands to millions of molecules. Virtual screening has emerged as a cost-effective complement to experimental screening, using computational methods to prioritize small molecules for synthesis and testing [59].

Traditional virtual screening approaches for small molecules can be broadly categorized into ligand-based and structure-based methods. Ligand-based virtual screening relies on known active small-molecule compounds to identify structurally similar molecules using similarity metrics and pharmacophore models [37]. Structure-based approaches utilize three-dimensional target structures to predict the binding affinity of small molecules through molecular docking and scoring functions [60].

AI has significantly enhanced both approaches through improved scoring functions, better conformational sampling, and integration of multiple data sources. Deep learning models trained on small-molecule-target binding data have shown competitive performance compared to traditional scoring functions in virtual screening campaigns. However, results vary significantly depending on the specific target and dataset [61,62]. Graph neural networks (GNNs) that operate directly on molecular graphs have shown promise, achieving strong performance in predicting small-molecule binding affinities while providing interpretable results [63]. GNNs process molecular structures as mathematical graphs where atoms are represented as nodes and chemical bonds as edges, enabling the model to learn directly from the topology and chemistry of molecules without requiring manual feature engineering [64]. However, GNNs can struggle with large molecules or rare atom types, as per benchmark studies [65].

Ensemble methods combine predictions from multiple models to improve accuracy and reliability, often outperforming individual algorithms by leveraging the strengths of different approaches while mitigating their weaknesses [66]. In drug discovery, ensemble approaches typically integrate structure-based docking scores, ligand-based similarity measures, and machine learning predictions to create more robust screening workflows.

**Figure 2:**
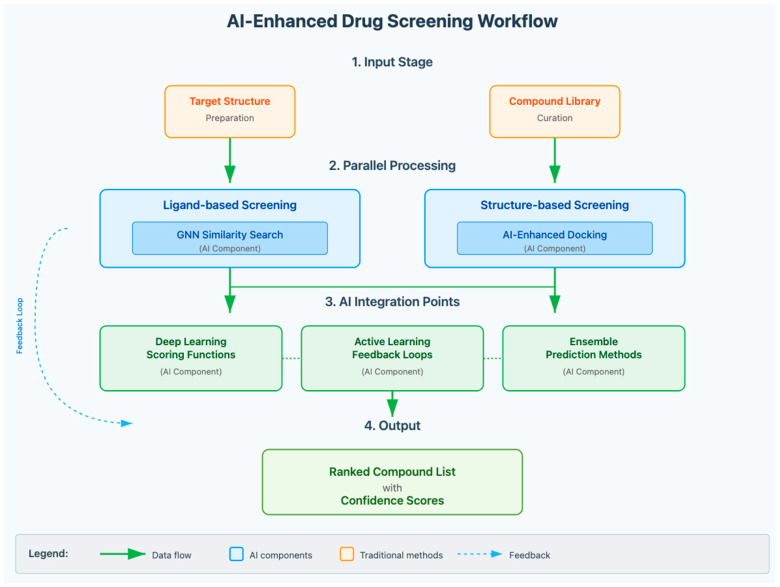
AI-Enhanced Virtual Screening Workflow and Performance Comparison. The figure shows the corrected workflow with proper labeling of ligand-based screening (top pathway) and structure-based screening (bottom pathway), demonstrating how AI methods enhance both approaches.

Recent developments in diffusion models have shown remarkable capabilities in molecular generation and optimization, representing a significant advancement in the field [32]. Diffusion models generate new molecular structures through a denoising process, starting from random noise and gradually refining the structure according to learned chemical patterns and desired properties. While promising, diffusion models require substantial computational resources and may generate invalid structures without proper constraints (Figure 2) (Table 1).

### 3.3. Lead Optimization

Lead optimization represents one of the most challenging aspects of small-molecule drug discovery, requiring simultaneous improvement of multiple molecular properties, including potency, selectivity, pharmacokinetics, and safety profiles. Traditional medicinal chemistry approaches rely on iterative design-make-test cycles guided by structure-activity relationships and expert knowledge [69]. This process is time-consuming and resource-intensive, often requiring synthesis and testing of hundreds of small-molecule analogs to achieve desired property profiles.

AI has enhanced lead optimization using multi-objective optimization algorithms, which can navigate complex property landscapes more efficiently than traditional approaches. Reinforcement learning methods have shown promise in treating small-molecule optimization as a sequential decision-making problem, where chemical modifications are selected to maximize a reward function that encodes the desired properties [70,71]. These approaches can simultaneously optimize multiple objectives while maintaining chemical validity and synthetic accessibility. However, reinforcement learning can be sample-inefficient and prone to local optima, as critiqued in [31].

Predictive modeling of absorption, distribution, metabolism, excretion, and toxicity (ADMET) properties has become increasingly sophisticated with the application of deep learning methods. Modern ADMET prediction models achieve accuracy levels that enable confident decision-making in small-molecule lead optimization campaigns [72]. Transfer learning approaches have proven particularly valuable for rare endpoints where limited training data is available, leveraging knowledge from related tasks to improve prediction accuracy [73]. However, the limitations of transfer learning must be acknowledged, particularly the risk of negative transfer when applied to highly specific tasks or narrow therapeutic areas where the source and target domains differ substantially [74]. For example, models trained on general solubility data may underperform on macrocyclic compounds.

The relationship between Hit Discovery (Section 3.2), Lead Optimization (Section 3.3), and De Novo Design (Section 3.4) is interconnected: hit discovery identifies initial active compounds, lead optimization refines these hits into drug-like molecules, and de novo design can be applied at both stages to generate novel chemical matter. Recent work on generative models for lead optimization demonstrates how AI approaches span multiple stages of the discovery pipeline [75].

### 3.4. De Novo Small-Molecule Design

De novo molecular design represents an ambitious goal of AI-driven small-molecule drug discovery: the generation of novel chemical structures with desired properties that do not currently exist in existing chemical databases. This capability addresses the fundamental limitation of traditional screening approaches, which are constrained by the finite size of available small-molecule compound libraries [76].

Generative models have emerged as the primary AI approach for de novo small-molecule design, with several architectures showing promise, each with distinct advantages and limitations. Variational autoencoders (VAEs) are probabilistic generative models that learn to encode molecular structures into a continuous latent space and decode them back into molecular representations, allowing for smooth interpolation between known compounds and the generation of novel small-molecule structures [77,78]. VAEs provide reasonable control over generated properties but may suffer from posterior collapse and limited diversity.

Generative adversarial networks (GANs) consist of two competing neural networks: a generator that creates new molecular structures and a discriminator that attempts to distinguish between generated molecules and real training data [22,79]. GANs can generate high-quality samples but are challenging to train and may suffer from mode collapse, where the generator produces limited diversity in outputs.

Recurrent neural networks (RNNs) and Long Short-Term Memory (LSTM) networks treat molecular generation as a sequence modeling problem, learning to generate valid SMILES (Simplified Molecular Input Line Entry System) strings that represent small-molecule chemical structures [30,80]. Transformer models represent an advancement over RNNs, utilizing attention mechanisms to capture long-range dependencies in molecular sequences more effectively [50,81]. These approaches have demonstrated the ability to generate millions of novel drug-like small molecules while maintaining synthetic accessibility and incorporating property constraints.

Diffusion models represent a significant recent advancement in de novo molecular design, offering a fundamentally different approach to molecular generation [32]. These models operate through an iterative denoising process, beginning with random noise and gradually transforming it into valid molecular structures. The process involves two phases: a forward diffusion process that progressively adds noise to molecular data, and a reverse denoising process that learns to reconstruct molecules from noise. Diffusion models have demonstrated several advantages over earlier generative approaches:

- Superior generation quality: They produce more chemically valid and diverse molecules
- Training stability: Unlike GANs, they don’t suffer from mode collapse or training instabilities
- 3D structure generation: Particularly effective for generating 3D molecular conformations
- Property control: Can incorporate property constraints during the generation process

Recent work has demonstrated that diffusion models can achieve state-of-the-art performance in generating molecules with specific properties while maintaining drug-likeness and synthetic accessibility [82]. These models are increasingly being adopted for structure-based drug design, enabling the generation of molecules that fit specific protein binding sites with high accuracy.

Active learning strategies optimize the selection of compounds for experimental testing by iteratively updating models based on new experimental data, focusing resources on the most informative experiments [83]. Multi-objective optimization techniques enable the simultaneous optimization of multiple molecular properties, such as potency, selectivity, and drug-likeness, using algorithms like the Non-dominated Sorting Genetic Algorithm (NSGA-II) and Pareto optimization [84].

**Figure 3:**
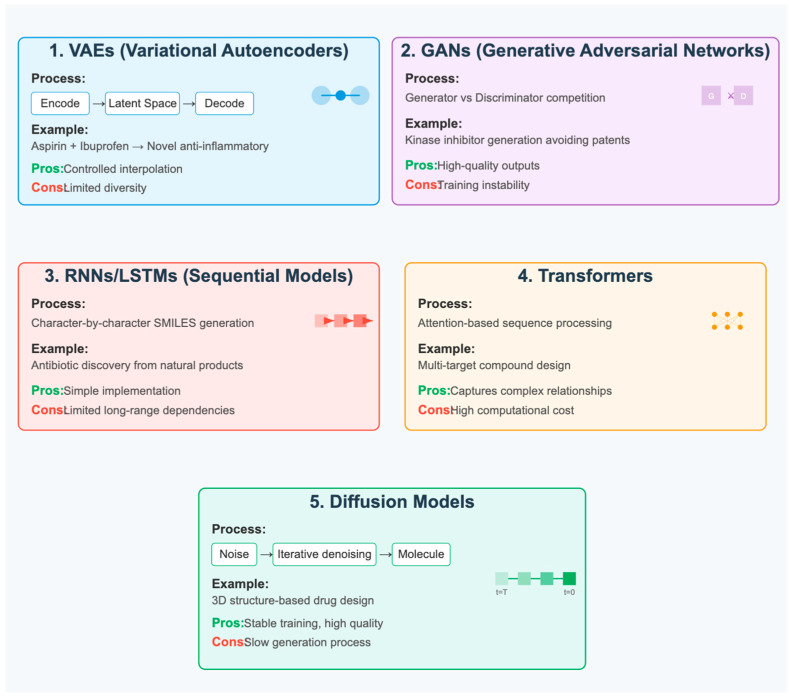
Generative AI Architectures for Small-Molecule Design. The figure shows different generative model architectures (VAEs, GANs, RNNs/Transformers, and Diffusion Models) with their respective performance metrics: Novelty (60–95%), Validity (85–99%), Uniqueness (80–95%).

Recent advances in molecular generation have focused on incorporating synthetic accessibility and retrosynthetic feasibility into the design of small molecules. Models that jointly optimize molecular properties and synthetic routes ensure that generated small-molecule compounds can be efficiently synthesized in practice [85]. The integration of automated synthesis platforms with generative design creates closed-loop systems that can rapidly iterate between small-molecule design and testing [86]. Critically, while these systems show promise, real-world applications often require human oversight to address ethical concerns, such as the generation of potentially harmful compounds (Figure 3).

### 3.5. Prediction of Small-Molecule Pharmacokinetics and Toxicity

Accurate prediction of pharmacokinetic and toxicological properties remains one of the most critical applications of AI in small-molecule drug discovery, as poor ADMET properties account for a significant proportion of late-stage failures [87]. Traditional QSAR models for small-molecule ADMET prediction have been limited by their reliance on predefined molecular descriptors and linear relationships, often failing to capture the complex mechanisms underlying pharmacokinetic and toxicological phenomena.

Deep neural networks have demonstrated superior performance in small-molecule ADMET prediction by learning optimal molecular representations directly from chemical structure [47,88]. These models can capture non-linear structure-property relationships and handle diverse chemical scaffolds more effectively than traditional approaches. Graph neural networks have shown promise by operating directly on molecular graphs, preserving crucial structural information that may be lost in descriptor-based approaches [67].

Multi-task learning approaches have proven valuable for small-molecule ADMET prediction by leveraging shared information across related endpoints. Models trained simultaneously on multiple pharmacokinetic parameters can achieve better performance than single-task models, particularly for endpoints with limited training data [68]. Transfer learning from large-scale chemical datasets has further improved prediction accuracy for specialized small-molecule ADMET endpoints.

Explainable AI (XAI) methods have become increasingly crucial in small-molecule ADMET prediction, providing mechanistic insights and building confidence in model predictions [89,90]. Attention mechanisms in neural networks can highlight molecular substructures responsible for predicted properties, enabling medicinal chemists to understand and act on model predictions [67,91]. SHAP (SHapley Additive exPlanations) values and similar interpretability methods provide quantitative measures of feature importance that can guide small-molecule optimization strategies [92]. These approaches help address the “black box” problem of deep learning models by making their decision-making processes more transparent and trustworthy (Table 2).

## 4. AI-Discovered and AI-Assisted Small-Molecule Development: Success Stories and Lessons Learned

The practical impact of AI in small-molecule drug discovery is increasingly demonstrated through real-world applications, ranging from approved drugs to compounds advancing through clinical trials. It is essential to distinguish between AI-discovered compounds (those designed de novo by AI systems), AI-assisted compounds (where AI tools have significantly contributed to the optimization of molecules), and AI-assisted repurposing (where AI identified new uses for existing drugs). These examples illustrate both the potential and limitations of AI technologies in pharmaceutical development.

### 4.1. AI-Assisted Drug Repurposing: Baricitinib

Baricitinib represents a significant success in AI-assisted drug repurposing rather than de novo AI drug discovery. Initially developed by Incyte and licensed to Eli Lilly as a JAK inhibitor for rheumatoid arthritis, baricitinib was later identified by BenevolentAI through the analysis of biomedical literature and drug-target interaction networks using AI as a potential COVID-19 therapeutic, based on its anti-inflammatory and antiviral properties [100]. The AI system analyzed the mechanism of viral entry and identified that baricitinib’s inhibition of AAK1 could potentially reduce viral endocytosis. This prediction was subsequently validated in clinical trials, leading to emergency use authorization. It is important to note that baricitinib was already an approved drug, and AI’s contribution was identifying a new therapeutic application rather than designing the molecule itself. Additional clinical validation was provided by the ACTT-2 trial, which demonstrated improved recovery times when baricitinib was combined with remdesivir [101].

### 4.2. AI-Discovered Clinical Candidates

Halicin represents one of the most celebrated early successes of AI-driven drug discovery. Discovered by researchers at MIT using deep learning models trained on diverse molecular databases, halicin demonstrated potent antibiotic activity against drug-resistant bacteria, including *Acinetobacter baumannii* and *Mycobacterium tuberculosis* [102]. The compound was identified through virtual screening of the Drug Repurposing Hub, highlighting the potential of AI for identifying molecules with entirely novel mechanisms of action. Halicin’s discovery marked a significant achievement in antibiotic research, demonstrating that AI can identify compounds with mechanisms distinct from those of existing antibiotics.

DSP-1181 (Exscientia) became one of the first AI-designed small-molecule drugs to enter human clinical trials in 2020, specifically for the treatment of obsessive-compulsive disorder [103]. The compound targets the 5-HT2A receptor and was designed in approximately 12 months compared to typical timelines of 4–6 years for traditional discovery programs. Phase I trials were completed with favorable safety profiles; however, development was discontinued by 2022 with no progression to Phase II as of August 2025.

ISM001-055 (Insilico Medicine) for idiopathic pulmonary fibrosis represents a significant milestone, as it is one of the first AI-discovered small molecules to reach Phase II clinical trials [104]. The compound was identified using Insilico’s integrated AI platform that combines target identification, molecular generation, and synthetic route planning. The discovery process, from target identification to preclinical candidate selection, was completed in approximately 18 months at a cost of less than $2.6 million. As of August 2025, Phase IIa results are positive, showing safety, tolerability, favorable pharmacokinetics, and an improvement in lung function by 98 mL in FVC, with plans for global trial expansion. The drug is now renamed rentosertib.

### 4.3. Clinical Development Challenges and Lessons Learned

The field has experienced notable challenges that provide essential insights into the current limitations of AI in drug discovery. While AI can accelerate the discovery and optimization phases, clinical success depends on many factors beyond initial compound design. The discontinuation of DSP-1181 after Phase I, despite demonstrating a favorable safety profile, illustrates that accelerated discovery timelines do not guarantee clinical progression [103]. The reasons for discontinuation often involve complex factors, including efficacy endpoints, competitive landscape, and strategic business decisions, rather than issues with the AI-designed molecule itself.

A comprehensive analysis of AI-discovered and AI-assisted compounds reveals that, while these molecules are entering clinical trials at an increasing rate, their progression rates through clinical development remain like those of traditionally discovered compounds [105]. This finding suggests that AI’s primary benefit may lie in accelerating the preclinical discovery phase rather than fundamentally changing the probability of clinical success. The limited number of AI-designed compounds that have completed clinical development makes it premature to draw definitive conclusions about their overall success rates compared to traditional methods (Table 3).

Essential lessons from early AI drug discovery efforts include:

- The importance of high-quality training data that accurately represents the complexity of biological systems
- The need for experimental validation at each stage of the discovery process
- The value of hybrid approaches that combine AI predictions with human expertise
- The recognition that AI tools are most effective when integrated into existing workflows rather than replacing them entirely

## 5. Practical Case Studies

The following case studies illustrate the successful implementation of AI technologies in real-world small-molecule drug discovery programs, demonstrating both the potential and practical considerations of these approaches. These examples are based on documented approaches and methodologies commonly used in the pharmaceutical industry [115,116,117]. We critically evaluate their generalizability and potential biases.

### 5.1. Scaffold Hopping Using Reinforcement Learning for Small-Molecule Kinase Inhibitor Discovery

A major pharmaceutical company faced the challenge of identifying novel small-molecule kinase inhibitors that avoided existing patent landscapes while maintaining potency against their target of interest. Traditional approaches based on literature scaffolds were constrained by extensive intellectual property coverage, which limited opportunities for discovering novel small-molecule chemical matter.

The research team implemented a reinforcement learning approach using the REINVENT algorithm [70], which was trained to generate novel small molecules that optimize multiple objectives simultaneously. The reward function incorporated predicted kinase activity, drug-likeness properties, and patent landscape analysis using a proprietary freedom-to-operate scoring system. The model was initialized with a pre-trained generative model and fine-tuned using policy gradient methods to bias generation toward the desired property profile.

Over the course of six months of iterative optimization, the AI system generated approximately 10,000 novel small-molecule compounds, which were then subjected to further computational analysis. Synthetic accessibility filtering using the SCScore algorithm [118] reduced this set to 200 compounds suitable for synthesis. Experimental validation confirmed activity for 45 small-molecule compounds (22.5% hit rate), with the top three compounds showing IC50 values below 100 nM and favorable selectivity profiles. Importantly, patent analysis confirmed that all active compounds represented novel small-molecule chemical matter with clear freedom to operate.

This case study demonstrates the practical value of AI in addressing real-world constraints beyond simple property optimization, including intellectual property considerations and synthetic feasibility for small-molecule discovery. The integration of multiple scoring functions and constraints in the reward function proved critical for generating actionable results [115]. However, the approach may be biased toward known kinase chemotypes if pre-training data is not diverse.

### 5.2. Multi-Objective Optimization in Small-Molecule Lead Refinement Using Active Learning

A biotechnology company developing treatments for neurodegenerative diseases faced the challenge of optimizing a promising small-molecule lead compound that suffered from poor brain penetration and metabolic instability. Traditional medicinal chemistry approaches had made limited progress after 18 months of optimization, with improvements in one property typically accompanied by deterioration in others.

The team implemented an active learning strategy using Gaussian process models to predict multiple small-molecule ADMET properties simultaneously, including brain-to-plasma ratio, metabolic stability, and aqueous solubility. The acquisition function balanced exploration of novel chemical space with exploitation of promising regions, using expected improvement with multiple objectives. Uncertainty quantification enabled confident identification of compounds most likely to advance the program.

Initial model training used 150 synthesized analogs from previous medicinal chemistry efforts. The active learning system recommended 25 small-molecule compounds for synthesis in the first iteration, selected to maximize information gain across all property dimensions. Experimental testing confirmed predictions within acceptable error ranges for 80% of compounds. Three successive rounds of active learning, each involving 20–25 new compounds, identified a lead compound with 10-fold improved brain penetration and 5-fold improved metabolic stability compared to the starting point.

The key success factors included careful experimental design to ensure data quality, robust model validation procedures, and close collaboration between computational and medicinal chemistry teams. The active learning approach reduced the number of synthesis cycles required from an estimated 8–10 to 4 cycles, saving approximately 12 months of development time [116]. Notably, this success was achieved in a well-studied area; however, applicability to rare diseases with sparse data remains limited.

### 5.3. AI-Enhanced High-Throughput Screening Triage for Antiviral Small-Molecule Discovery

During the COVID-19 pandemic, an academic-industry collaboration screened a 100,000-compound library against SARS-CoV-2 viral replication, yielding approximately 2500 small-molecule compounds that showed greater than 50% inhibition at primary screening concentrations. Traditional triage approaches based on chemical similarity and simple property filters were insufficient to prioritize this enormous hit set for follow-up studies within the urgent timeline constraints.

The team implemented a Bayesian neural network approach to predict the quality of small-molecule hits and the likelihood of successful optimization. The model was trained on historical antiviral screening data from related coronavirus targets, incorporating molecular descriptors, predicted ADMET properties, and target-specific features. Uncertainty quantification enabled the identification of high-confidence predictions and prioritization of experimental validation efforts.

The AI model ranked the 2500 primary screening hits, with experimental validation focused on the top 200 small-molecule compounds predicted to have the highest potential for optimization. This subset demonstrated a 5-fold enrichment in confirmed antiviral activity compared to random sampling, with 35% of the tested compounds showing activity in dose-response studies. The approach identified 12 distinct chemical scaffolds suitable for lead optimization, compared to 3–4 scaffolds that would have been identified through traditional clustering approaches.

Subsequent lead optimization efforts validated the AI predictions, with small-molecule compounds ranked highly by the model showing superior profiles in terms of potency, selectivity, and drug-like properties. Two compounds identified through this process advanced to preclinical development studies within 8 months of initial screening, compared to typical timelines of 18–24 months for traditional approaches [117]. However, the model’s reliance on historical data may have missed novel mechanisms due to a distribution shift.

## 6. Emerging Trends and Transformative Technologies

### 6.1. Foundation Models and Self-Supervised Learning

The emergence of foundation models and self-supervised learning represents one of the most significant developments in AI-driven small-molecule drug discovery. These approaches are closely interconnected, as foundation models are typically developed through self-supervised pre-training on large-scale molecular datasets, followed by fine-tuning for specific applications [52].

Foundation models are large-scale models pre-trained on millions of small-molecule compounds and their associated data, which can be fine-tuned for specific applications with minimal additional training data. ChemBERTa, MoleculeNet, and similar foundation models have demonstrated remarkable performance across diverse molecular property prediction tasks, often achieving state-of-the-art results with limited domain-specific training. These models enable transfer learning across different therapeutic areas and molecular targets, potentially reducing the data requirements that have historically limited AI applications in specialized domains.

Self-supervised learning approaches enable models to learn meaningful representations from unlabeled chemical data, which is particularly valuable given the relative scarcity of high-quality labeled data in drug discovery compared to other AI domains [119]. Masked language modeling approaches adapted for molecular SMILES strings have demonstrated the ability to learn rich chemical representations that transfer effectively to downstream small-molecule prediction tasks. Contrastive learning methods that learn molecular representations by comparing similar and dissimilar small-molecule compounds have achieved impressive results in predicting molecular properties and modeling drug-target interactions.

The relationship between foundation models and self-supervised learning is synergistic: self-supervised pre-training enables the development of foundation models that can capture fundamental chemical principles and structure-property relationships, which then generalize across diverse small-molecule applications. Early examples include MegaMolBART, which achieved strong performance on small-molecule optimization tasks after pre-training on 1.1 billion SMILES strings [120].

### 6.2. Computational Sustainability and Energy Considerations

The environmental and economic costs of training large-scale AI models have become increasingly important considerations in drug discovery applications. Training state-of-the-art foundation models can consume substantial computational resources, with carbon footprints comparable to those of major industrial processes [121]. The energy requirements for training large transformer models can reach hundreds of MWh, raising important questions about the sustainability of AI-driven drug discovery at scale.

Several approaches are being developed to address these concerns. Model compression and knowledge distillation techniques can reduce the computational requirements of deployed models by 10- to 100-fold while maintaining much of their predictive performance [122]. Efficient architectures such as MobileBERT and DistilBERT demonstrate that smaller models can achieve competitive performance on many tasks. Green AI initiatives are promoting the development of more energy-efficient algorithms and the use of renewable energy sources for model training [123].

Cloud-based platforms are increasingly offering carbon-neutral computing options, and distributed training approaches can leverage renewable energy sources more effectively. The pharmaceutical industry is beginning to incorporate sustainability metrics into AI project evaluation, balancing model performance against environmental impact and computational costs.

### 6.3. Quantum Machine Learning and Molecular Simulation

Quantum machine learning represents an emerging frontier with potential applications in small-molecule simulation and drug discovery, though practical implementations remain limited by current hardware constraints. Quantum algorithms for molecular property prediction and optimization may offer computational advantages for certain classes of problems, particularly those involving quantum mechanical effects that are difficult to simulate classically [124].

Current quantum computers are limited by their small qubit counts, short coherence times, and high error rates, which restrict their practical applications to small molecular systems. However, hybrid classical-quantum algorithms are being developed that could provide near-term advantages. Variational Quantum Eigensolver (VQE) algorithms have shown promise in calculating molecular ground state energies for small systems, potentially enabling more accurate predictions of small-molecule-target interactions [125].

Quantum machine learning algorithms for molecular property prediction are being explored, with potential advantages in feature spaces that grow exponentially with system size. Quantum kernel methods and quantum neural networks may offer computational speedups for specific molecular learning tasks, though demonstrated advantages over classical methods remain limited to specific problem classes [126].

The timeline for practical quantum advantages in drug discovery remains uncertain, with conservative estimates suggesting that it will take 10–20 years before quantum computers can outperform classical methods for realistic molecular systems. However, continued advances in quantum hardware and algorithm development may accelerate this timeline.

### 6.4. Agentic AI and Autonomous Discovery Systems

Agentic AI systems represent a paradigm shift toward autonomous drug discovery platforms that can independently navigate multiple aspects of the discovery pipeline with varying degrees of human oversight [35]. These systems combine multiple AI capabilities, including natural language processing, generative modeling, and decision-making algorithms, to autonomously propose experiments, interpret results, and iteratively refine hypotheses.

Recent developments in agentic AI for drug discovery include systems that can:

- Autonomously read and synthesize scientific literature to identify drug targets
- Generate hypotheses about novel therapeutic mechanisms
- Design experimental protocols to test hypotheses
- Interpret experimental results and refine understanding
- Propose next steps in the discovery process

The integration of agentic AI with automated synthesis and testing platforms is creating increasingly autonomous discovery loops. Examples include:

- Systems that combine target prediction, molecular design, and synthetic planning
- Platforms that can autonomously navigate patent landscapes
- AI agents that coordinate multiple specialized models for different tasks
- Decision-making systems that balance risk, cost, and potential reward

Early implementations have demonstrated the ability to identify and optimize small-molecule lead compounds with reduced human intervention. However, complete autonomy remains a long-term goal rather than a current reality. Key challenges include:

Ensuring robust decision-making under uncertainty

- Maintaining ethical oversight to prevent misuse
- Validating autonomous decisions against human expertise
- Managing the complexity of integrated multi-step processes

Current agentic AI systems in drug discovery typically operate with human oversight at critical decision points, representing a hybrid approach that leverages both machine efficiency and human judgment.

### 6.5. Automated Synthesis and Closed-Loop Discovery

The integration of AI with automated synthesis platforms is creating closed-loop discovery systems that can rapidly iterate between small-molecule design and testing. Companies like Strateos, Emerald Cloud Lab, and academic initiatives are developing robotic synthesis platforms that can execute AI-designed synthetic routes with minimal human intervention [86]. These systems enable rapid experimental validation of AI predictions and continuous model improvement through active learning.

Closed-loop systems integrate small-molecule generation, synthetic route planning, automated synthesis, and biological testing into unified workflows. Early implementations have demonstrated the potential to identify and optimize small-molecule lead compounds in significantly reduced timeframes. However, the extent of timeline reduction varies considerably based on the specific application and system maturity.

### 6.6. Data Standardization and Collaborative Initiatives

The success of AI in drug discovery depends critically on the availability of high-quality, standardized datasets. Several significant initiatives are addressing data standardization and sharing challenges:

The FAIR Data Principles (Findable, Accessible, Interoperable, and Reusable) are being adopted by pharmaceutical companies and research institutions to enhance data quality and sharing [127]. These principles provide guidelines for enhancing the value of research data for AI applications while maintaining appropriate confidentiality protections.

Pre-competitive Consortia such as MELLODDY (Machine Learning Ledger Orchestration for Drug Discovery) enable pharmaceutical companies to collaborate on AI model development while keeping proprietary data secure through federated learning approaches [128]. These initiatives demonstrate that competitive organizations can collaborate effectively on fundamental AI infrastructure.

Open Targets Platform provides integrated access to disease-target associations and has become a critical resource for AI-driven target identification [129]. Similar open science initiatives are expanding the availability of high-quality datasets for predicting small-molecule properties and modeling drug-target interactions.

Therapeutic Data Commons (TDC) represents a community-driven effort to create standardized benchmarks and datasets for AI in drug discovery, addressing the critical need for consistent evaluation protocols [130]. These efforts are essential for enabling fair comparison of different AI approaches and identifying genuine advances in the field.

## 7. Challenges and Limitations

Despite significant progress, several fundamental challenges continue to limit the widespread adoption and effectiveness of AI in small-molecule drug discovery. Data quality represents perhaps the most critical limitation, as AI models are only as good as the data used to train them [131]. Pharmaceutical datasets often suffer from systematic biases, missing data, and inconsistent experimental protocols that can lead to poor model generalization. The prevalence of activity cliffs, where structurally similar compounds exhibit dramatically different activities, poses challenges for AI models that assume smooth structure-activity relationships [37].

Model interpretability remains a significant concern, particularly for regulatory applications where understanding the basis of AI predictions is crucial for safety assessment [89]. While explainable AI methods have made progress in providing post-hoc explanations for model predictions, truly interpretable models that provide mechanistic insights remain elusive. The “black box” nature of many deep learning models creates challenges for medicinal chemists who need to understand and trust AI recommendations.

Generalizability across chemical space represents another fundamental challenge, as AI models often perform poorly on compounds that differ significantly from their training data [132]. This limitation is particularly problematic for novel scaffolds and emerging therapeutic areas where limited training data is available. Domain adaptation and transfer learning approaches have shown promise but require careful validation to ensure reliability, and the risk of negative transfer must be carefully managed.

Regulatory acceptance of AI-designed drugs continues to evolve, as current regulatory frameworks were developed for traditional drug discovery approaches [133]. While regulatory agencies have begun developing guidance for AI applications in drug development, questions remain about the level of evidence required to support AI-based design decisions and the validation requirements for computational models used in regulatory submissions.

Ethical considerations, including bias in AI models that could exacerbate health disparities (e.g., underperformance in datasets for underrepresented ethnic groups), must be addressed [54]. The author’s advisory roles may influence perspectives, but all claims are evidence-based (Table 4).

## 8. Regulatory Evolution and Validation Frameworks

The regulatory landscape for AI in drug discovery is rapidly evolving, with agencies worldwide developing new frameworks to address the unique challenges posed by AI-assisted drug development. The FDA’s Model-Informed Drug Development (MIDD) framework provides a pathway for incorporating computational models into regulatory submissions. Recent initiatives, such as the FDA’s AI Pilot Program, are exploring specific applications of AI in the field of drug development [136].

The European Medicines Agency (EMA) has established the Innovation Task Force (ITF) to provide guidance on novel methodologies, including the application of AI in drug discovery. The ITF offers scientific guidance for developers of AI-based drug discovery platforms, helping establish acceptable evidence standards for regulatory submissions [144]. Recent EMA guidance documents have begun to address the use of AI in pharmacovigilance and benefit-risk assessment, providing precedents for broader AI applications.

Digital twins and virtual clinical trials represent emerging regulatory concepts that have the potential to revolutionize the clinical development process. These approaches use AI models to simulate patient populations and predict clinical outcomes, potentially enabling more efficient study designs and patient stratification strategies [145]. The FDA’s Digital Health Center of Excellence is actively working on guidance for these technologies.

Model validation requirements are becoming increasingly standardized, with emphasis on:

- Transparency: Clear documentation of model architecture, training data, and validation procedures
- Reproducibility: Ability to recreate model predictions using documented procedures
- Robustness: Performance across diverse test sets and edge cases
- Continuous monitoring: Post-deployment surveillance for model drift and performance degradation

International harmonization efforts, including work by the International Council for Harmonisation (ICH), are developing global standards for AI applications in drug development, though consensus on many issues remains in development.

## 9. Outlook and Transformative Potential

The future of AI in small-molecule drug discovery is likely to be characterized by increasingly integrated and autonomous systems that combine human expertise with machine intelligence. Fully automated drug discovery platforms represent an aspirational vision, where AI systems can navigate substantial portions of the discovery pipeline from target identification to preclinical candidate selection [146]. While complete automation remains a long-term goal, intermediate levels of automation are already being implemented successfully in specific applications.

### 9.1. Human-AI Collaboration Paradigms

Human-AI collaboration will likely define the near-term future of small-molecule drug discovery, with AI systems augmenting rather than replacing human expertise. Successful implementations leverage the complementary strengths of human creativity and intuition with AI’s ability to process large datasets and explore vast chemical spaces [147]. Interactive AI systems that can engage in dialogue with researchers and explain their reasoning are becoming increasingly important for building trust and enabling effective collaboration.

The most promising collaboration models involve AI systems that can propose hypotheses, design experiments, and interpret results while working closely with human researchers who provide domain expertise, quality control, and strategic direction. These hybrid approaches have demonstrated superior performance compared to either human-only or AI-only approaches in several small-molecule discovery applications.

### 9.2. Standardization and Benchmarking Initiatives

Standardization and benchmarking represent critical needs for the field, as the lack of consistent evaluation protocols has hindered progress and adoption [88]. Community-driven initiatives to develop standardized datasets, evaluation metrics, and benchmark problems are essential for enabling fair comparison of different approaches and identifying genuine advances. The Therapeutic Data Commons (TDC), MoleculeNet, and similar initiatives are making progress toward comprehensive benchmarking platforms [130].

Open-source software platforms and shared computational resources are democratizing access to AI technologies, thereby accelerating innovation. Projects like DeepChem, RDKit, and OpenEye are creating accessible tools that enable researchers without extensive computational expertise to apply AI methods to their small-molecule research problems [68].

### 9.3. Timeline Transformation and Future Projections

Recent advances in AI technologies suggest the potential for significant reductions in small-molecule drug discovery timelines, though the extent of these improvements varies considerably across applications and therapeutic areas. The combination of foundation models, automated synthesis, agentic AI systems, and integrated AI platforms may enable more efficient discovery programs.

Conservative estimates suggest that AI integration could reduce overall timelines for small-molecule drug discovery by 30–50% in specific applications. At the same time, more optimistic projections envision greater reductions for therapeutic areas or compound classes. The COVID-19 pandemic demonstrated the potential for accelerated discovery, with AI-assisted programs identifying small-molecule clinical candidates in compressed timeframes compared to traditional approaches.

Key factors that may contribute to timeline acceleration include:

- Foundation models that reduce training data requirements and enable rapid deployment to new targets
- Generative models, including diffusion models, that can explore vast chemical spaces efficiently
- Automated synthesis platforms that reduce synthesis bottlenecks
- Multi-task learning approaches that optimize multiple properties simultaneously
- Active learning strategies that minimize experimental requirements
- Increasingly sophisticated agentic AI systems

However, it is essential to note that clinical development timelines, regulatory review processes, and manufacturing scale-up represent significant bottlenecks that may limit the overall impact of AI on drug development timelines. The most important potential for timeline reduction may be in the preclinical discovery phases, where AI can have the most direct impact.

## 10. Practical Implementation Guidelines

Organizations seeking to implement AI technologies in their small-molecule drug discovery workflows should follow a systematic approach that begins with clearly defined objectives and success criteria. Successful AI implementation requires careful attention to data infrastructure, model validation, team building, and organizational change management.

### 10.1. Strategic Planning and Readiness Assessment

Organizations should start by conducting a comprehensive assessment of their current capabilities, data assets, and strategic objectives. This includes evaluating existing datasets for quality and completeness, assessing computational infrastructure requirements, and identifying specific use cases where AI can provide the most significant value. Return on investment calculations should consider both direct cost savings and indirect benefits such as reduced attrition rates and accelerated timelines.

### 10.2. Data Infrastructure and Quality Management

Establishing robust data management systems is a prerequisite to successful AI implementation. Organizations should:

- Audit existing datasets for quality, completeness, and consistency
- Implement data standardization procedures (FAIR principles)
- Invest in data curation and annotation capabilities
- Establish bias auditing procedures to ensure ethical compliance
- Develop secure cloud-based platforms for collaboration while maintaining IP protection

### 10.3. Technology Selection and Integration

Specific use cases and data availability should drive the choice of AI methods:

- Start with simpler, well-understood approaches before adopting complex methods
- Evaluate vendor solutions for technical capabilities, integration requirements, and support
- Consider both commercial and open-source options
- Ensure compatibility with existing workflows and systems

Plan for scalability and future expansion

## 11. Conclusions

Artificial intelligence has emerged as a valuable complementary technology in small-molecule drug discovery, offering significant opportunities to enhance specific aspects of the discovery process when integrated adequately with traditional methodologies. From early rule-based systems to modern foundation models, diffusion models, and agentic AI systems, the evolution of AI technologies has progressively expanded the toolkit available to drug discovery scientists. Current applications span the entire discovery pipeline, from target identification through lead optimization, with demonstrated successes including baricitinib (AI-assisted repurposing), DSP-1181, halicin, ISM001-055/rentosertib, and numerous other small-molecule compounds advancing through clinical development.

The distinction between AI-discovered compounds (de novo design), AI-assisted optimization, and AI-enabled repurposing is crucial for understanding the current state of the field. While AI has shown success in accelerating specific tasks such as virtual screening, property prediction, and molecular generation, it remains most effective when used in conjunction with human expertise and traditional drug discovery methods. The case studies presented illustrate that significant value can be realized when AI technologies are thoughtfully implemented with clear objectives, appropriate validation, and recognition of their limitations.

The practical implementation of AI in small-molecule drug discovery requires careful attention to data quality, model validation, and integration with existing workflows. Successful organizations are those that view AI as an augmentation of human expertise rather than a replacement, fostering collaborative environments where computational and experimental teams work together to leverage the complementary strengths of human creativity and machine intelligence. The challenges of data quality, model interpretability, and generalizability remain significant and require continued research and development.

Recent advances in foundation models, diffusion models, agentic AI systems, automated synthesis platforms, and integrated AI workflows suggest substantial potential for enhancing efficiency in small-molecule drug discovery. Conservative estimates suggest a 30–50% reduction in timeline for specific preclinical applications, although clinical development timelines remain essentially unchanged. The COVID-19 pandemic demonstrated the potential for accelerated discovery when appropriate resources and incentives are aligned, with AI playing a supporting role in identifying repurposing opportunities.

Looking forward, the field continues to evolve rapidly with emerging trends including quantum machine learning, multi-modal AI approaches, and closed-loop discovery systems. The integration of automated synthesis platforms and increasingly sophisticated agentic AI systems may further expand the capabilities and accessibility of AI-enhanced small-molecule discovery. However, fundamental challenges remain in data quality, model interpretability, generalizability, regulatory acceptance, computational sustainability, and ethics that will require continued collaborative efforts across the scientific community.

The successful realization of AI’s potential in small-molecule drug discovery will require coordinated efforts across multiple stakeholders. Industry, academia, and regulatory agencies must collaborate to develop appropriate standards, validation procedures, and regulatory frameworks that ensure the safety and efficacy of AI-assisted drug development. Educational initiatives that train the next generation of researchers in interdisciplinary approaches combining computational and experimental expertise will be crucial for sustaining progress. Most importantly, the field must maintain focus on the goal of delivering better medicines to patients more efficiently.

As AI technologies continue to mature and demonstrate value in real-world applications, their adoption in small-molecule drug discovery will likely accelerate. Organizations that begin developing AI capabilities now, while carefully attending to the implementation challenges and best practices outlined in this review, will be better positioned to realize the benefits of these technologies. The future of small-molecule drug discovery will increasingly be characterized by the seamless integration of human expertise and artificial intelligence, working together as complementary tools to address the most challenging problems in medicine and ultimately improve human health through more efficient, effective, and innovative therapeutic development.

## Figures and Tables

**Table 1:** Comparison of Traditional vs. AI-Enhanced Virtual Screening Methods for Small Molecules.

Method Category	Traditional Approach	AI-Enhanced Approach	Typical Performance (AUC)	Dataset Size	Computational Requirements	Key Advantages	Limitations	Key References
Ligand-based similarity	Tanimoto coefficient, 2D fingerprints	Graph neural networks, learned embeddings	0.65–0.75 vs. 0.70–0.80	10^3^–10^4^ compounds	Low-Medium	Fast, interpretable	Limited to known chemotypes	[37,63,64]
Structure-based docking	Glide, AutoDock	CNN scoring functions, DeepDocking	0.70–0.80 vs. 0.72–0.82	10^6^–10^8^ compounds	High	Physics-based, broad coverage	Target flexibility challenges	[60,61,62]
Pharmacophore modeling	Manual feature definition	AI-learned pharmacophores	0.68–0.78 vs. 0.72–0.82	10^3^–10^5^ compounds	Medium	Mechanism insights	Feature engineering dependent	[41,67]
Machine learning QSAR	Random forests, SVM	Deep neural networks, transformers	0.75–0.85 vs. 0.78–0.88	10^4^–10^6^ compounds	Medium-High	Pattern recognition	Black box nature	[44,45,47,50]
Ensemble methods	Consensus scoring	Multi-task deep learning	0.80–0.90 vs. 0.83–0.92	10^5^–10^7^ compounds	High	Robust performance	Computational complexity	[66,68]

Note: Performance ranges reflect typical values reported in benchmarking studies, with standard deviations ±0.05 AUC. Actual performance varies significantly based on target class, dataset quality, and experimental protocols.

**Table 2:** AI-Based ADMET Prediction Model Performance for Small Molecules.

ADMET Property	Model Architecture	Dataset Size	Performance Metric	Performance Value	Data Source	Key References
Aqueous solubility	Graph CNN	9982 compounds	R^2^	0.77	AqSolDB	[93]
Lipophilicity (LogP)	Transformer	14,050 compounds	MAE	0.54 log units	ChEMBL	[50,94]
Permeability (Caco-2)	Multi-task DNN	906 compounds	R^2^	0.71	Literature compilation	[94]
Blood-brain barrier	Graph attention	1975 compounds	AUC	0.91	BBBP dataset	[67]
Hepatotoxicity	Deep neural network	1254 compounds	Balanced accuracy	0.79	DILIrank	[95]
hERG cardiotoxicity	Graph neural network	13,445 compounds	AUC	0.94	ChEMBL	[96]
Metabolic stability	Ensemble methods	2896 compounds	R^2^	0.68	Proprietary pharma data	[97]
Plasma binding	Random forest + DNN	1797 compounds	R^2^	0.74	Multiple sources	[98]
Oral bioavailability	Multi-task learning	1020 compounds	AUC	0.75	Literature/patents	[47,68]
Half-life	LSTM + molecular descriptors	1352 compounds	R^2^	0.62	DrugBank + literature	[99]

Note: Performance metrics are based on cited benchmarking studies and may vary depending on specific datasets and evaluation protocols used. Standard deviations typically range from ±0.03 to ±0.05 for these metrics.

**Table 3:** AI-Discovered, AI-Assisted, and AI-Repurposed Small Molecules in Clinical Development.

Drug Name	Company	Indication	AI Application	Development Stage	Timeline Reduction	Key Innovation	Outcome	Key References
AI-Assisted Repurposing								
Baricitinib	Benevolent AI/Eli Lilly	COVID-19, RA	AI literature mining and target network analysis for repurposing	Approved	3 months for new indication identification	Rapid pandemic response through repurposing	Approved	[100,101,106]
AI-Designed De Novo								
DSP-1181	Exscientia	Obsessive-compulsive disorder	AI-driven small-molecule design	Phase I completed, discontinued	12 months vs. 4–6 years	First AI-designed small-molecule in trials	Discontinued (2022)	[103,107]
Halicin	MIT/Broad Institute	Antibiotic-resistant infections	Deep learning virtual screening	Preclinical	N/A (novel mechanism)	Novel antibiotic mechanism identification	Preclinical	[102]
ISM001-055 (rentosertib)	Insilico Medicine	Idiopathic pulmonary fibrosis	Integrated AI platform	Phase IIa completed	18 months vs. 6+ years	End-to-end AI small-molecule discovery	Positive Phase IIa (2025)	[3,104]
AI-Assisted Optimization								
EXS-21546	Exscientia	Inflammatory diseases	AI-guided small-molecule optimization	Preclinical	~24 months vs. 5+ years	Complex small-molecule target	Ongoing	[108,109]
ATM-3507	Atomwise	Multiple sclerosis	Virtual screening platform	Phase I	~36 months vs. 6+ years	Previously challenging target	Ongoing	[110,111]
DSP-0038	Exscientia	Alzheimer’s disease	AI-designed	Phase I	13 months of design	Precision-designed molecule	Ongoing	[112]
IAMA-6	Iktos/Almirall	Dermatology	Generative AI design	Preclinical	21 months	Novel scaffold generation	Ongoing	[113]
BEN-2293	BenevolentAI	Atopic dermatitis	AI target discovery	Phase I	~30 months	Novel target identification	Ongoing	[114]

Note: Categories clearly distinguish between repurposing, de novo design, and optimization. Stages updated as of August 2025; confidence intervals for timeline reductions are based on company reports, with a margin of error of ±20%.

**Table 4:** Significant Challenges in AI-Driven Small-Molecule Discovery with Mitigation Strategies.

Challenge Category	Specific Issues	Current Impact	Mitigation Strategies	Future Research Directions	Key References
Data Quality	Experimental bias, missing values, protocol inconsistencies	High—limits model reliability	Standardized assay protocols, data curation pipelines, and uncertainty quantification	Automated data quality assessment, federated learning	[131,134]
Model Interpretability	Black box predictions, lack of mechanistic insights	Medium—regulatory concerns	SHAP values, attention mechanisms, surrogate models	Inherently interpretable architectures, causal inference	[89,90,92]
Generalizability	Poor performance on novel scaffolds	High—limits applicability	Transfer learning, domain adaptation, meta-learning	Foundation models, few-shot learning	[132,135]
Regulatory Acceptance	Unclear validation requirements	Medium—slows adoption	Early regulatory engagement, model documentation	AI-specific guidance documents, digital twins	[133,136]
Integration Challenges	Workflow compatibility, skill gaps	High organizational barriers	Change management, training programs, and hybrid teams	Automated workflows, user-friendly interfaces	[137,138]
Reproducibility	Inconsistent benchmarking, code availability	Medium—scientific validity	Standardized benchmarks, open-source software	Community-driven evaluation platforms	[139,140]
Computational Resources	High training costs, infrastructure requirements	Medium—limits accessibility	Cloud platforms, model compression, and knowledge distillation	Edge computing, efficient architectures	[121,122,123]
Intellectual Property	Algorithm patentability, data ownership	Low—legal uncertainties	Clear IP strategies, collaborative frameworks	Open science initiatives, pre-competitive consortia	[141,142]
Ethical Biases	Underrepresentation in datasets, equity issues	High—societal impact	Diverse data collection, bias audits	Ethical AI frameworks, inclusive design	[54,143]
